# Successful limb salvage beyond the golden time following blunt traumatic open complete transection of the femoral artery and vein in a patient with cardiac arrest: a case report

**DOI:** 10.1186/s40792-021-01264-x

**Published:** 2021-08-04

**Authors:** Hoshi Himura, Kenichiro Uchida, Masahiro Miyashita, Yasumitsu Mizobata

**Affiliations:** Department of Traumatology and Critical Care Medicine, Graduate School of Medicine, Osaka City University, 1-5-7 Asahimachi, Abeno-ku, Osaka City, 545-8586 Japan

**Keywords:** Femoral artery transection, Blunt trauma, Vascular injury, Extremity vessel injury, Revascularization

## Abstract

**Background:**

Open complete transection of the femoral artery and vein following blunt trauma is extremely rare. Furthermore, even if the patient has been successfully resuscitated, it is sometimes difficult in most patients to preserve the injured limb, especially after damage control resuscitation. We report a case of open complete transection of the femoral artery and vein secondary to high-energy blunt trauma and a successful limb preservation treatment strategy.

**Case presentation:**

A 57-year-old Asian man was transferred to hospital after having fallen from the 15th floor of a condominium. The patient was in cardiac arrest at the scene, but was successfully resuscitated by emergency medical services staff. On arrival, the patient’s hemodynamics were completely collapsed with active external bleeding from the thigh, so we immediately started resuscitation including activation of massive transfusion protocol and temporarily ligated the transected proximal superficial femoral artery, deep femoral artery just distal after branching lateral femoral circumflex artery and the superficial femoral vein. Following radiological findings showing a potential pelvic fracture with active bleeding, we also performed retroperitoneal packing in the resuscitation room and moved the patient to the angiography room for transcatheter arterial embolization. The patient’s consciousness was preserved and perfusion of the injured limb was barely maintained after his hemodynamics were adequately stabilized. As we detected weak perfusion of the lower limb via a potential collateral flow from the lateral femoral circumflex artery branches from deep femoral artery by pulse doppler of the dorsal pedis artery, we decided to reconstruct superficial femoral artery and vein at 24 h after injury using great saphenous vein bypass grafts. The patient was transferred to a rehabilitation hospital with good neurological and limb outcome after hospitalization for 52 days.

**Conclusion:**

We successfully preserved the patient’s lower limb after cardiac arrest and complete transection of the femoral artery and vein and achieved a good neurological outcome. Even if a femoral artery needs to be ligated temporarily, careful observation and assessment should be performed so as not to lose the chance to salvage the limb even during damage control resuscitation.

## Introduction

Open transection of femoral artery and vein is rare and commonly lethal [1–4]. Although an abbreviated damage control strategy for major vessel injury such as a temporary shunt or ligation can be administered effectively [5, 6], it is sometimes difficult to preserve the injured limb especially in multiple trauma patients with significant hemodynamic instability [2, 3].

## Case presentation

A 57-year-old Asian man without relevant previous medical history was hospitalized following a fall from the 15th floor of a condominium. A witness said he had fallen from the condominium and had contacted electrical wires before hitting the ground. Prior to being transferred to the hospital, when the emergency medical service personnel arrived on scene, the patient had no pulse, but spontaneous circulation returned after 4 min of chest compression. On hospital arrival, the patient’s airway was maintained spontaneously, respiratory rate was 30 breaths/min, blood pressure was unmeasurable but the left femoral artery pulse was weakly palpable, heart rate was regular at 136 beats/min, body temperature was 36.1 °C, and Glasgow Coma Scale was E3V1M5. His right inner thigh was lacerated halfway around, and he was actively bleeding from this site. Blood analysis data on admission are shown in Table 1. We immediately initiated a massive transfusion protocol, secured the airway of the patient with endotracheal intubation and temporarily ligated the proximal exposed superficial femoral artery (SFA), deep femoral artery (DFA) just distal part after branching lateral femoral circumflex artery (LFCA), and superficial femoral vein separately. As we could not detect backflow from the distal artery, possibly because of intimal injury, spasm, or thrombus, and also as there was no time to expose this artery, we could not temporarily shunt it. As well, a tourniquet could not be applied because the injury site was too proximal to the torso. In parallel with preparations to initiate resuscitative endovascular balloon occlusion of the aorta via the left femoral artery, we packed the thigh and sequentially searched for other sites of bleeding because of the patient’s continued hemodynamic instability. Although we found no other sites of bleeding according to an extended focused assessment with sonography for trauma examination, subsequent plain pelvic radiography suggested the potential for retroperitoneal bleeding with iliac fracture. Considering the patient’s hemodynamics, we performed retroperitoneal packing by making suprapubic incision and entering preperitoneal space comprising the para-vesical and presacral space and then packing pads were placed.

**Table 1:** Blood analysis data on admission

Analyzed items	Value	Reference normal range (lower limit–upper limit)
White blood cell count (/µL)	11.000	4300 to 8000
Platelet count (× 10^4^/µL)	29.6	18.0 to 34.0
C-reactive protein (mg/dL)	0.21	0 to 0.4
Creatinine (mg/dL)	1.06	0.5 to 1.1
Blood urea nitrogen (mg/dL)	20	8 to 20
Creatine kinase (IU/L)	296	59 to 248
Lactate dehydrogenase (IU/L)	421	124 to 222
Total *bilirubin* (mg/dL)	0.3	0.2 to 1.0
Fibrinogen (mg/dL)	378	200 to 400
Fibrin degradation products (µg/mL)	96.9	0 to 10.0
Antithrombin III (%)	74	70 to 120
Prothrombin time-international normalized ratio	1.20	0.90 to 1.10
pH	7.269	7.35 to 7.45
Base excess	− 12.7	− 7 to 2
Lactate level (mmol/L)	10.6	0.5 to 1.6

**Fig. 1:**
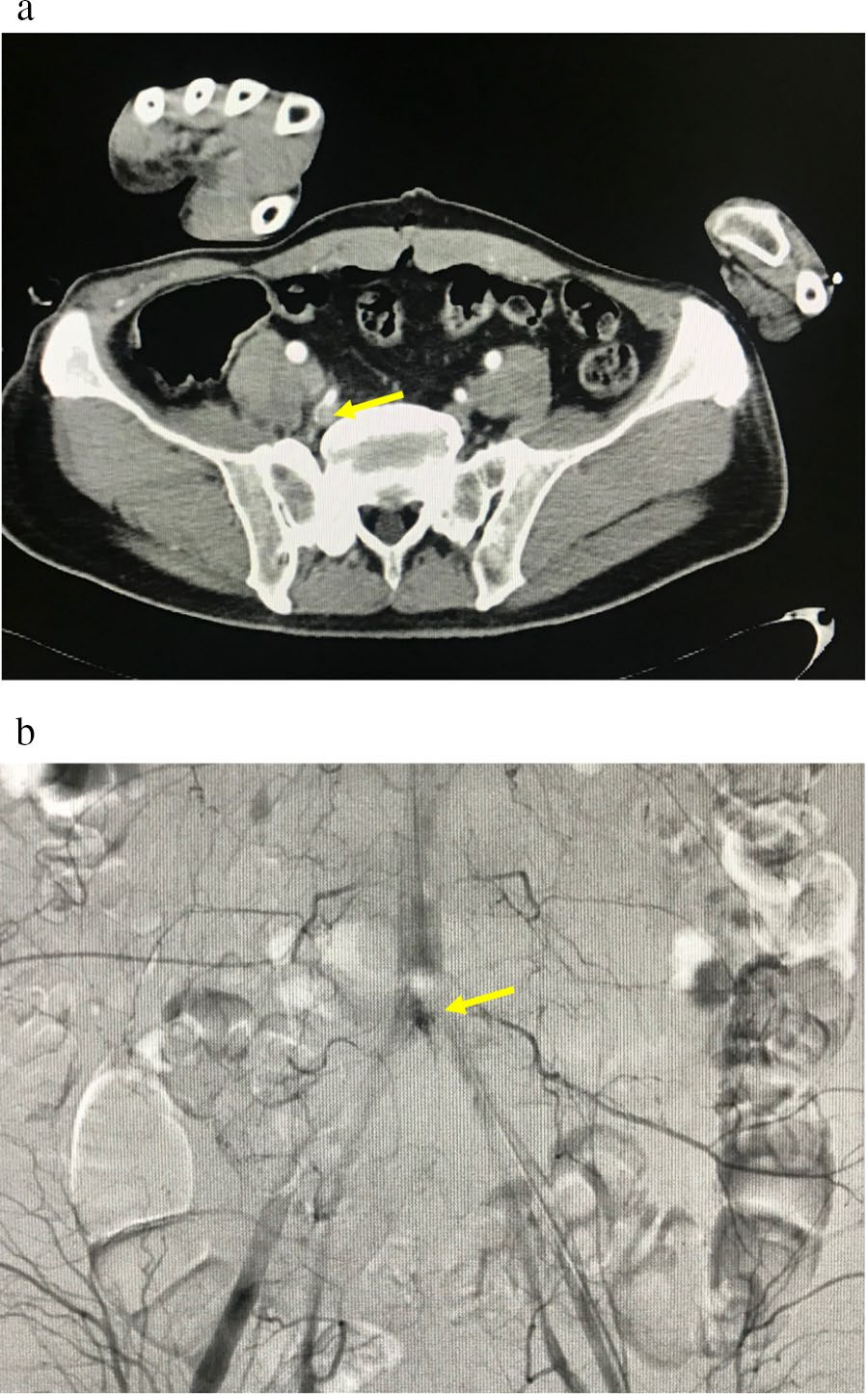
Contrast-enhanced computed tomography and X-ray images on admission. **a** Fractures of the 4th lumbar vertebra and right iliac body concomitant with arterial extravasation (yellow arrow) were detected. **b** We performed transcatheter arterial embolization to control this bleeding (yellow arrow)

**Fig. 2:**
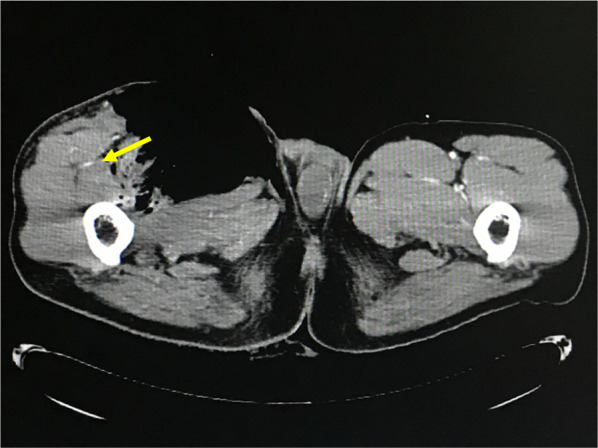
Contrast-enhanced computed tomography image on admission. The right superficial and deep femoral artery were interrupted. However, lateral femoral circumflex artery branches from deep femoral artery seemed patent (yellow arrow). Further examination of distal vessels could not be done because of the need to prioritize resuscitation

**Fig. 3:**
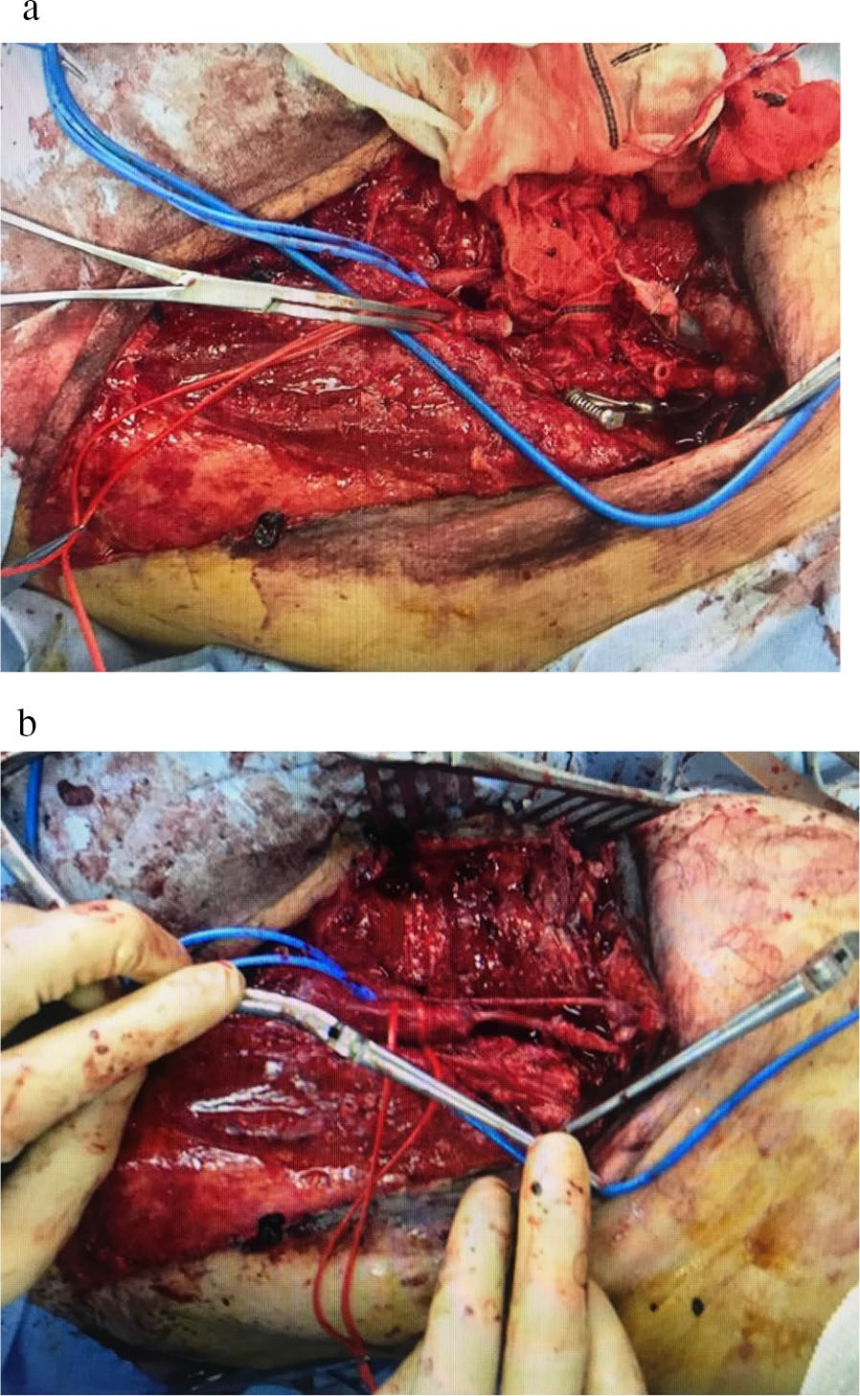
Photographs before (**a**) and after (**b**) reconstruction of the superficial femoral artery and vein with great saphenous vein bypass grafts

After this was completed, the patient was stable enough to undergo contrast-enhanced computed tomography (CECT). CECT showed fractures of the 4th lumbar vertebra and right iliac body concomitant with arterial extravasation at this site (Fig. 1a), so we performed transcatheter arterial embolization (Fig. 1b). Although CECT showed that the right SFA and DFA just distal part after branching LFCA were interrupted (Fig. 2), narrow blood flow in the distal popliteal artery seemed to be maintained by collateral perfusion from LFCA as pulse doppler detected a weak pulse in the dorsal pedis artery. The ankle brachial pressure index at this time was approximately 0.54. After confirming good consciousness of the patient and resolving resuscitation-related conditions such as hypothermia and acidosis, we decided to perform revascularization of the femoral artery and vein by bypassing them with great saphenous vein grafts at approximately 24 h after the initial injury. The operation was performed with the patient in the supine position. Minor fresh thrombus was obtained from distal side of SFA and SFV by Fogarty catheter insertion and we could obtain sufficient back flow from both vessels. We decided to sacrifice the DFA and vein and bypass the SFA. The great saphenous vein was harvested from the left limb, and a graft of about 7 cm in length was anastomosed to the intact intima of the SFA (Fig. 3a). As the superficial femoral vein also appeared to be bypassable, we bypassed it with a great saphenous vein (Fig. 3b). All anastomoses were performed by use of 6-0 monofilament suture with the parachute technique.

The soft tissue was also severely injured with some loss of tissue apparent. We initially considered covering the anastomosed vessels with viable muscle, but as the tissue deficit was not completely uncoverable, negative pressure wound therapy was applied over the muscle tissue.

**Fig. 4:**
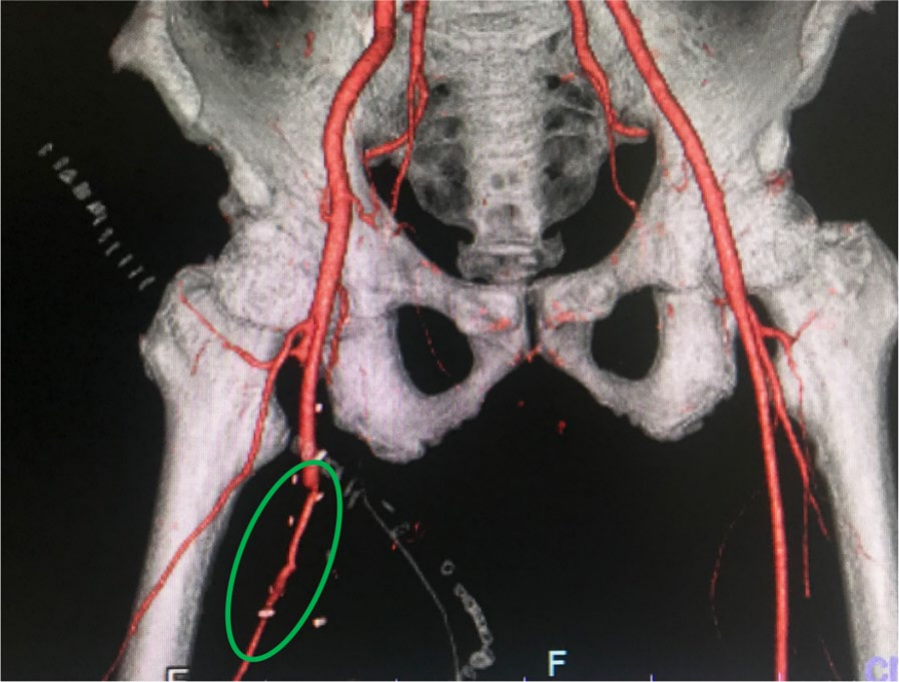
Contrast-enhanced computed tomography image obtained 8 days after surgery showed the patency of blood flow through the reconstructed femoral vessels (green oval)

The patient was extubated on the 4th hospital day, and musculocutaneous grafting was performed 22 days after the initial operation following completion of negative pressure wound therapy. Postoperative CECT showed the patency of flow through the femoral vessels (Fig. 4) and the score of ABI improved 1.15. As adjuvant therapy, although we could not administrate anticoagulant drugs prior to surgery on the basis of concomitant injury, systemic unfractionated heparin infusion was used during and post 10 days after bypass surgery. And then we converted to oral administration of warfarin and controlled the international normalized ratio of prothrombin time at the range of 2.0–2.5.

The patient was transferred to a rehabilitation hospital 52 days after admission without any symptom such as swelling or intermittent claudication.

## Discussion

Open total transection of major vessels mostly occurs with penetrating injury, and mortality and morbidity remain high especially in hemodynamically unstable patients [2–4]. Femoral vessel injury is one of the common major extremity vessel injuries and reportedly accounts for approximately 70% of total extremity vessel injuries [7].

The first priority in the treatment strategy for major extremity vessel injury is bleeding control, and recently, two temporary options have been devised especially for hemodynamically unstable patients. First is the application of a temporary shunt. This technique is quite effective in maintaining distal tissue perfusion with minimum invasiveness and minimum time required [8–10]. However, if the vessel is completely transected secondary to a blunt injury mechanism, as in the present patient, prompt adequate exposure of the distal vessel is sometimes not easy and can be time consuming. In our patient, who was injured by falling from a height, we had to rapidly survey his other injuries and sites of bleeding causing hemodynamic instability. Second is the application of a tourniquet. Currently, the efficacy of a tourniquet to control extremity bleeding is commonly described as resulting in good outcomes [5, 6, 11–13]. However, we could not apply it due to the proximal site of the injury.

The golden time for revascularization of the extremities is considered to be within 6 to 8 h after the injury [14–17], but CECT and pulse Doppler examinations fortunately revealed collateral perfusion to the injured distal lower limb. Hence, even though the Mangled Extremity Severity Score of our patient was 12 [18], we carefully observed and assessed limb perfusion by Doppler and prioritized resuscitation of the patient. Although we bypassed the femoral vessels approximately 24 h after the injury, no reperfusion-related morbidities or signs of compartment syndrome were observed as recently reported elsewhere [14, 19]. Even if there is no choice but to ligate the femoral vessels in an abbreviated surgery, the patient should be resuscitated from the viewpoint of careful assessment of whether the limb can be salvaged.

## Conclusion

We successfully salvaged our patient’s lower limb even after resuscitation from cardiac arrest and complete transection of the femoral artery and vein. Even if a femoral artery needs to be ligated temporarily, subsequent careful observation and assessment should always be performed so as not to lose the chance to salvage the limb.

## Data Availability

Data sharing is not applicable to this article as no data sets were generated or analyzed for the study.

## Abbreviations

CECT: Contrast-enhanced computed tomography
DFA: Deep femoral artery
SFA: Superficial femoral artery
LFCA: Lateral femoral circumflex artery
