# Correction to: Successful limb salvage beyond the golden time following blunt traumatic open complete transection of the femoral artery and vein in a patient with cardiac arrest: a case report

**DOI:** 10.1186/s40792-021-01280-x

**Published:** 2021-08-27

**Authors:** Hoshi Himura, Kenichiro Uchida, Masahiro Miyashita, Yasumitsu Mizobata

**Affiliations:** Department of Traumatology and Critical Care Medicine, Graduate School of Medicine, Osaka City University, 1-5-7 Asahimachi, Abeno-ku, Osaka City, 545-8586 Japan

## Correction to: Surg Case Rep (2021) 7: 177 https://doi.org/10.1186/s40792-021-01264-x

Following publication of the original article [1], the authors identified an error in the family name of the third author Masahiro Miyashita.

The incorrect author name is: Masahiro Hiyashita.

The correct author names is: Masahiro Miyashita.

The author group has been updated above and the original article has been corrected in the author list and Authors’ contributions section.
